# Effects of the administration of Shinbaro 2 in a rat lumbar disk herniation model

**DOI:** 10.3389/fneur.2023.1044724

**Published:** 2023-03-10

**Authors:** Won Kyung Kim, Joon-Shik Shin, Jinho Lee, Wonil Koh, In-Hyuk Ha, Hyen Joo Park, Sang Kook Lee, Jin Young Hong

**Affiliations:** ^1^College of Pharmacy, Natural Products Research Institute, Seoul National University, Seoul, Republic of Korea; ^2^Jaseng Hospital of Korean Medicine, Seoul, Republic of Korea; ^3^Jaseng Spine and Joint Research Institute, Jaseng Medical Foundations, Seoul, Republic of Korea

**Keywords:** lumbar disc herniation, Shinbaro 2, pro-inflammatory cytokines, inflammation, disk degeneration-related factors

## Abstract

The current standard for the pharmacological management of lumbar disk herniation (LDH), involving non-steroidal anti-inflammatory drugs, muscle relaxants, and opioid analgesics, often carries a risk of adverse events. The search for alternative therapeutic options remains a vital objective, given the high prevalence of LDH and the critical impact on the quality of life. Shinbaro 2 is a clinically effective herbal acupuncture against inflammation and various musculoskeletal disorders. Therefore, we explored whether Shinbaro 2 exerts protective effects in an LDH rat model. The results showed that Shinbaro 2 suppressed pro-inflammatory cytokines, interleukin-1 beta, tumor necrosis factor-alpha, disk degeneration-related factors, matrix metalloproteinase-1,−3,−9, and ADAMTS-5 in LDH rats. Shinbaro 2 administration reinstated a behavioral activity to a normal level in the windmill test. The results indicated that Shinbaro 2 administration restored spinal cord morphology and functions in the LDH model. Therefore, Shinbaro 2 exerted a protective effect in LDH *via* actions on inflammatory responses and disk degeneration, indicating that future research is warranted to assess the action mechanisms further and validate its effects.

## 1. Introduction

Lumbar disk herniation (LDH) is a musculoskeletal disorder where an intervertebral disk of the lumbar spine is herniated, with or without tearing of the annulus fibrosus *via* degeneration or external force (1). Depending on the volume and region of herniation, the herniated disk often compresses the spinal cord or nerve root, provoking neurological symptoms (2). Clinical manifestations of LDH typically include radicular pain and loss of sensation and motor function in the affected regions, which is usually accompanied by lower back pain (3). Rather than one single cause, multiple factors contribute to the pathogenesis of LDH, including aging, genetic factors, nutrition, toxic factors, metabolic disorders, low-grade infection, neurogenic inflammation, autoimmunity, and mechanical factors (4). Physical compression of the nerve and subsequent biochemical stimuli resulting from the herniated disk are among the components underlying the pathological mechanisms of LDH (5). After herniation, pro-inflammatory cytokines such as tumor necrosis factor-alpha (TNF-α), interleukin-1 beta (IL-1β), and IL-6 are released, and the expression of catabolic molecules such as syndecan-4 and matrix metalloproteinases (MMPs) are elevated (6, 7). Thus, the extracellular matrix is degraded, enabling the infiltration of recruited immune cells (8). Together with prostaglandin (PG) expression, the activation of the acid-sensing ion channel 3 and transient receptor potential cation channel subfamily V member 1 contributes to the pain sensation (9). LDH treatment can be clinically divided into surgical and conservative treatments (10). Clinical studies have shown that conservative treatment for low back and radiation pain helps alleviate functional and clinical symptoms in patients with LDH (11). The therapeutic effect has been scientifically proven to be effective or safe *via* experimental studies. Shinbaro 2 (Jaseng Hospital prescription) is a type of pharmacopuncture developed from medicinal herbal substances used for GCSB-5 (shinbaro^®^) standardized by the Korea Food and Drug Administration (KFDA) and has anti-inflammatory, neuro-protective, cartilage-regenerative, and pain control effects (12, 13). GCSB-5 comprises six wild herbs, including *Cibotium barometz, Bang-Poong (Saposhnikovia divaricata (Turcz) Schischkin), Eucommia ulmoides, Ogapi* (*Acanthopanax sessiliflorum, Achyranthes japonica*, and *Glycine max*), and Shinbaro 2 is prepared by adding four herbs *(Ostericum koreanum, Angelica pubescens, Paeonia albiflora*, and *Scolopendra subspinipes)* to five medicinal herbs (*Cibotium barometz, Saposhnikovia divaricata, Eucommia ulmoides, Acanthopanax sessiliflorus*, and *Achyranthes japonica*) of GCSB-5 (14). Although the main chemical components of Shinbaro 2 have not been studied, many studies have reported the constituents and pharmacological activities of each material of Shinbaro 2. Previous studies have reported the anti-inflammatory and antioxidant effects of *Eucommia ulmoides* (15) and *Acanthopanax sessiliflorum* (16). Effects of *Achyranthes japonica* with regard to anti-inflammatory and anti-osteoporotic properties have also been reported (17). *Scolopendra subspinipes mutilans* has traditionally been used for the treatment of arthritis and neuropathic diseases such as lower back pain and peripheral injury, and various experimental approaches have demonstrated its ethnopharmacological activities against inflammatory diseases (18). *Cibotium barometz* has been reported to have osteoclastogenesis inhibitory efficacy and bone-strengthening activity in the treatment of lumbago, rheumatism, sciatica, and osteoporosis (19). In addition, among the four herbs added to Shinbaro 2, *Ostericium koreanum* plays an important role in providing anti-inflammatory properties through the downregulation of prostaglandin E2 and nitric oxide. It has also been reported to have anti-cancer, neuroprotective, gastrointestinal, and cardioprotective effects (20). The pharmacological effects of the other three herbs that are included in Shinbaro 2 also exhibit biological activities such as anti-cancer, anti-inflammatory, antioxidant, antibiotic, and analgesic activities (21–24). In particular, previous studies on the effects of GCSB-5 and Shinbaro 2 in disk diseases, showed that GCSB-5 administration reduced mechanical allodynia and improved radicular pain through the downregulation of the neuroglial activity in the spinal dorsal horn and the DRG expressions of calcitonin gene-related peptide (CGRP) and transient receptor potential vanilloid 1 (TRPV1) (25). Shinbaro 2 can effectively suppress the rat lumbar spinal stenosis (LSS)-induced increase in pain and behavior disorders, which is similar to the symptoms observed in patients with real LSS by attenuating pro-inflammatory cytokines and inflammatory mediators (12, 26). Therefore, Shinbaro 2 has been widely used in herbal acupuncture to treat musculoskeletal conditions, alleviating lumbar degenerative disease (LDD) with LSS and LDH. The clinical effects of Shinbaro 2 treatment have been validated, and the therapeutic findings of clinical trials were reported (27). Although Shinbaro 2 was included in major treatment guidelines for Korean medicine doctors and recommended as a pharmacopuncture treatment for patients with LDD, the pathophysiological mechanism underlying this effect is not entirely understood. In the present study, we aimed to provide a scientific rationale for the use of Shinbaro 2 based on the rapidly emerging requirement for evidence-based treatment. Using behavioral, histological, and molecular analyses, we investigated whether Shinbaro 2 enhances functional recovery *via* MMP-9 and MMP-13 inhibitory and anti-inflammatory effects in an LDH rat model. To the best of our knowledge, this is the first study to date to demonstrate the *in vivo* effect of Shinbaro 2 administration, thus scientifically verifying the therapeutic effect for LDH.

## 2. Materials and methods

### 2.1. Preparation of Shinbaro 2

A total of nine herbal medicines were commercially prepared and authenticated by Dr. J. Lee, Jaseng Hospital of Korean Medicine, Seoul, Korea. Briefly, nine dried wild herbs [*Cibotium barometz* rhizome (0.0013 g/mL), the root of *Saposhnikovia divaricate* (0.0013), the stem bark of *Eucommia ulmoides* (0.0013), the stem and root of *Acanthopanax sessiliflorus* (0.0013), rhizomes and roots of *Ostericum koreanum* (0.0013), the root of *Angelica pubescens* (0.0013), the root of *Achyranthes japonica* (0.0013), *Scolopendra subspinipes* (0.0013), and the root of *Paeonia albiflora* (0.0027)] were boiled in 70% ethanol for 3 h and then freeze-dried to a powder form. The powder was filtered, sterilized, and dissolved in distilled water to prepare the various concentration for administration in LDH rats.

### 2.2. *In vivo* model of lumbar disk herniation

All animal use and care followed the guidelines approved by the Seoul National University Institutional Animal Care and Use Committee (IACUC; permission number: SNU201610-2). Male Sprague Dawley rats (8 weeks old, 280–300 g) were purchased from Central Laboratory Animal, Inc. (Seoul, Korea) and housed in the animal care facility at Seoul National University under pathogen-free conditions with a 12 h light-dark schedule. Rats were anesthetized with Zoletil (Virbac, 50 mg/kg, intraperitoneal). Laminectomies were conducted by a midline dorsal incision to expose the left L5 nerve roots and associated dorsal root ganglion (DRG). Autologous nucleus pulposus was harvested from the coccygeal vertebra (Co2-3) of each tail and applied to the left L5 nerve roots just proximal to the DRG (28). In the normal group, the surgical process was identical except for the implantation of the nucleus pulposus.

### 2.3. Shinbaro 2 intramuscular or oral administration

Shinbaro 2 was administered once daily for 3 weeks starting after surgery in two different ways: (i) *via* the intramuscular route and (ii) *via* the oral route. The rats were divided into eight groups (*n* = 6/group): normal group (laminectomy without LDH), control group (LDH only), ILS-2 (LDH + intramuscular Shinbaro 2 of weight 2 mg/kg), ILS-10 (LDH + intramuscular Shinbaro 2 of weight 10 mg/kg), ILS-20 (LDH + intramuscular Shinbaro 2 of weight 20 mg/kg), OS-20 (LDH + oral Shinbaro 2 of weight 20 mg/kg), OS-200 (LDH + oral Shinbaro 2 of weight 200 mg/kg), and E2-10 (LDH + subcutaneous 17β-estradiol of weight 10 μg/kg). The body weights of all rats were monitored for up to 2 weeks and sacrificed at 3 weeks for histological and molecular analyses.

### 2.4. Windmill test

To evaluate the coordination of motor functions and spontaneous motility, we assessed rat balance utilizing running wheels in a windmill test. The rotational speed of the activity wheel accelerated from four revolutions per minute (rpm) to 40 rpm over 5 min (Ugo Basile 47700, Comerio, Italy). The rats were trained to run on the activity wheel daily for 3 days before inducing surgical LDH. The normal, control, and Shinbaro 2 groups were examined for the number of walking steps in the activity cage. The number of walking steps was measured before (0 day) and at 2, 7, 10, and 14 days after LDH. Each animal was tested three times each day, and an average value was obtained.

### 2.5. ELISA assay

Blood samples were centrifuged at 1,500 rpm for 10 min. The collected supernatant from the serum samples was frozen at −70°C until subsequent analysis. Serum levels of PGE_2_, TNF-α, and IL-1β were measured with the corresponding ELISA kits (R&D Systems, Minneapolis, MN, USA). All experiments were conducted according to the manufacturer's instructions.

### 2.6. Histological analysis

For hematoxylin and eosin (H&E) staining, the spinal specimen containing the intervertebral disk and spinal cord at the L5 level was fixed in 10% neutral buffered formalin at 4°C for 24 h and then decalcified in 20% formic acid for 3 days. Decalcified samples were washed with water for 24 h to remove the remaining formalin, gradually dehydrated in 50–100% alcohol, and substituted with xylene. After paraffin infiltration and embedding, the tissue samples were sliced at a thickness of 5 μm using a rotary microtome (Leica Biosystems, Buffalo Grove, IL, USA). Tissue slides were de-paraffinized with xylene and also 100 and 50% alcohol. The sectioned tissues were rinsed with PBS and immersed in hematoxylin solution for 2 min. After washing with tap water for 2 min, the sections were stained in eosin for 10 s, dehydrated through a graded ethanol series, cleared with xylene, mounted, and placed under a cover slip. The sections stained with H&E were observed with an optical microscope (Carl Zeiss, Jena, Germany).

### 2.7. Real-time PCR

Extracted tissue samples were obtained from the L5 level in each group. The tissues were then subjected to an RNA extraction kit (Active Motif, Carlsbad, CA, USA) to isolate RNA from the samples. To validate the purity of the sample and calculate the RNA and protein contents, the optical density was measured at 260 nm and 280 nm, respectively. In general, 1 μg of total RNAs was analyzed using real-time PCR. The samples were reacted with reverse transcriptase from the avian myeloblastosis virus (Promega, Madison, WI, USA) and oligo dT primer of the gene of interest, first at 42°C for 60 min, then heated to 99°C for 5 min, and finally placed on ice to deactivate the transcriptase enzyme. The synthesized cDNA was a linear strand. cDNA samples (5 μL) diluted 50 times were added to the primer (Table 1) and iQ™ SYBR^®^ Green Supermix (Bio-Rad, Hercules, CA) to reach a total volume of 20 μL. Real-time PCR was conducted with the samples using the MiniOpticon system (Bio-Rad, Hercules, CA, USA). Threshold cycle (CT) values indicate the number of cycles performed when the amount of the target gene reaches the threshold; the CT value of each sample was calculated and analyzed using the comparative CT method with MJ Opticon Monitor software (MFR information; Bio-Rad, Hercules, CA, USA).

**Table 1:** Primer sequences used for real-time PCR.

**Gene**	**5^′^-3^′^**	**Primer sequence**
iNOS	Forward	ACCATGGAGCATCCCAAGT
	Reverse	CAGCGCATACCACTTCAGC
IL-1β	Forward	TGTGATGAAAGACGGCACAC
	Reverse	CTTCTTCTTTGGGTATTGTTTGG
TNF-α	Forward	AGTTGGGGAGGGAGACCTT
	Reverse	CATCCACCCAAGGATGTTTAG
MMP-9	Forward	CCTCTGCATGAAGACGACATAA
	Reverse	GGTCAGGTTTAGAGCCACGA
MMP-13	Forward	TCGCATTGTGAGAGTCATGCCAACA
	Reverse	TGTGGTTCCAGCCACGCATAGTCA
ADAMTS-5	Forward	AGAGTCCGAACGAGTTTACG
	Reverse	GTGCCAGTTCTGTGCGTC

### 2.8. Western blot

Protein samples were isolated from the collected spinal tissue using a protein extract kit (Active Motif, Carlsbad, CA, USA). Samples were quantified to 30–50 μg and electrophoresed in an 8–10% sodium dodecyl sulfate-polyacrylamide gel at 100 V for 2.5 h. Gels containing the protein of interest were cut and subjected to electro-transfer onto a polyvinylidene difluoride membrane (Millipore, Bedford, MA, USA) for 1 h. The membrane was washed twice with PBS containing 0.1% Tween 20 in PBST and then blocked with 5% non-fat dry milk at room temperature for 1 h. After washing with PBST, the membranes were reacted with primary antibodies against the proteins of interest (iNOS, IL-1β, TNF-α, MMP-9, MMP-13, ADAMTS-5, and β-actin) diluted in the ratio 1:1,000–1:2,000 in PBST with 2.5% bovine serum albumin at 4°C for 12 h. After washing with PBST, the membranes were reacted with HRP-conjugated secondary antibodies (1:1,500–1:2,000) at room temperature for 2 h. The membranes were then washed three times with PBST and incubated with enhanced chemiluminescence western blotting detection reagents (Lab Frontier, Suwon, Korea). The generated luminescence was detected and analyzed using a LAS 4000 system (Fuji Film Corp., Tokyo, Japan).

### 2.9. Statistics

All results were statistically confirmed using Prism software (GraphPad, San Diego, CA) as the means ± standard error of the mean. Multiple comparisons among five groups were analyzed *via* one-way and two-way analysis of variance with Tukey's *post-hoc* test. Differences were considered statistically significant if *p* < 0.001 vs. normal group and ^*^p < 0.05, ^**^p < 0.01, ^***^p < 0.001, and ^****^p < 0.0001 vs. the control group.

## 3. Results

### 3.1. Body weight, behavior, and histological assessments

**Figure 1:**
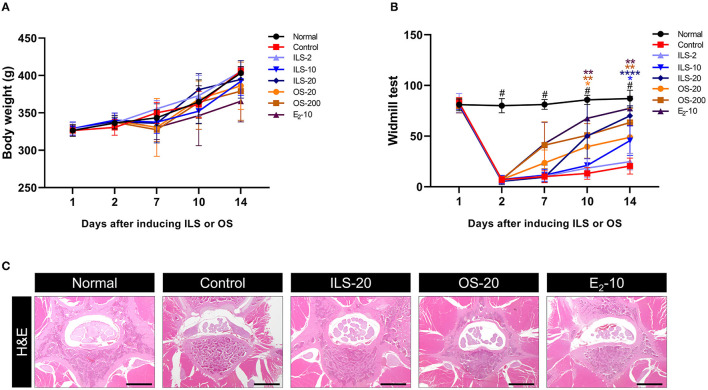
Body weight, behavior, and histological assessments. **(A)** Body weight curve of each group for 14 days. **(B)** Behavioral assessment of each group using the windmill test for 14 days. **(C)** H&E-stained images of the spinal structure following the administration of Shinbaro 2 with two routes in LDH rats. Scale bar = 200 μm. Data are expressed as mean ± SEM. Significant differences indicated as having ^#^a *p* < 0.001 compared vs. the blank group, ^*^a *p* < 0.05, ^**^a *p* < 0.01, and ^****^a *p* < 0.0001 vs. the control group were analyzed *via* one-way ANOVA with Tukey's *post-hoc* test.

To evaluate the safety of Shinbaro 2, we first confirmed the change in body weight for up to 2 weeks after the intramuscular and oral administration of various doses of Shinbaro 2 to LDH rats. The body weights showed an increasing trend with time but did not differ significantly between the groups (Figure 1A). The functional recovery was observed in the windmill activity cage during the two-week experimental period. When LDH was surgically induced, the behavioral activity was significantly lower than that of the normal group. The behavioral activity improved more in LDH rats administered with Shinbaro 2 than that in the control group 10 days after administration. The oral route showed more a rapid functional recovery than the intramuscular route for Shinbaro 2 administration to LDH rats. However, there was no statistical difference between the administration route (Figure 1B). We also confirmed the morphological changes using H&E staining. The normal group showed a spinal cord with an intact spinal canal; however, the control group exhibited a deformed intervertebral disk, suggesting nerve root compression. Therefore, the Shinbaro 2 intramuscular or oral administration and the E_2_ positive control group led to the structural recovery of the LDH-induced damage toward a normal morphology (Figure 1C).

### 3.2. Effect of Shinbaro 2 on the expression of serum PGE_2_, IL-1β, and TNF-α levels in LDH rats

**Figure 2:**
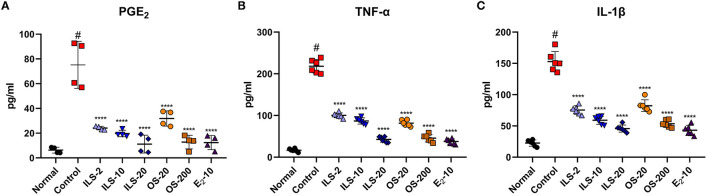
Effect of Shinbaro 2 on the expression of serum PGE2, IL-1β, and TNF-α levels in LDH rats. **(A–C)** ELSA for **(A)** PGE2, **(B)** TNF-α, and **(C)** IL-1β levels in the serum of each group at 14 days. Data are expressed as the means ± SEM. Significant differences indicated as ^#^a *p* < 0.001 compared vs. the blank group and ****a *p* < 0.0001 vs. the control group were analyzed *via* one-way ANOVA with Tukey's *post-hoc* test.

We explored the anti-inflammatory effect of Shinbaro 2 on the serum cytokines of PGE_2_, TNF-α, and IL-1β related to systemic inflammation in LDH rats. Serum PGE_2_ was significantly higher in the control group than in the normal group, as determined by using an enzyme immunoassay. However, Shinbaro 2 administration significantly lowered LDH-associated serum PGE_2_ levels in a dose-dependent manner, regardless of the administration route similar to the E2 positive control (Figure 2A). In addition, we confirmed the pro-inflammatory cytokines, TNF-α, and IL-1β in the serum. The IL-1β cytokine sends downstream signals, inducing the expression of various inflammatory genes. TNF-α and IL-1β were significantly higher in the control group than that in the normal group. Meanwhile, Shinbaro 2 significantly reversed the LDH-associated increase in the protein expression of TNF-α and IL-1β in a dose-dependent manner (Figures 2B, C), which were similar to those of the E2 level. Therefore, Shinbaro 2 was effective in reducing the inflammatory response in LDH rats.

### 3.3. Effect of Shinbaro 2 on the expression of iNOS, IL-1β, TNF-α mRNA, and protein in LDH rats

**Figure 3:**
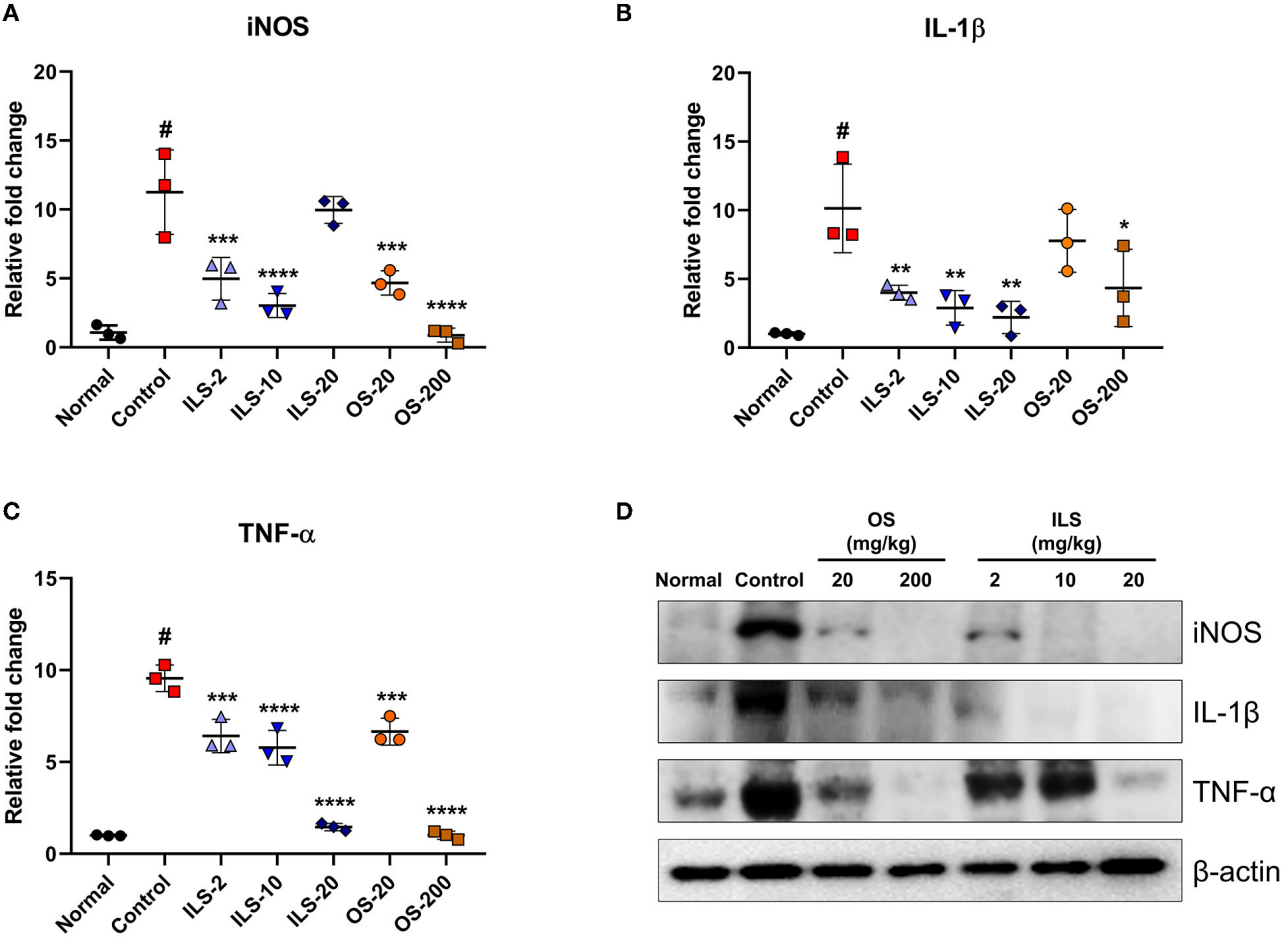
Effect of Shinbaro 2 on the expression of iNOS, IL-1β, TNF-α mRNA, and protein in LDH rats. **(A–C)** Gene expressions analyzed with tissue samples after Shinbaro 2 administration with two routes in LDH rats for 14 days: **(A)** *iNOS*, **(B)** *IL-1*β, **(C)** *TNF-*α, and **(D)** western blot analysis of iNOS, IL-1β, and TNF-α protein expression at 14 days. Data are expressed as mean ± SEM. Significant differences indicated as ^#^A *p* < 0.001 compared vs. the blank group, *a *p* < 0.05, **a *p* < 0.01, ***a *p* < 0.001, and ****a *p* < 0.0001 vs. the control group were analyzed *via* one-way ANOVA with Tukey's *post-hoc* test.

The *iNOS* level in the spinal cord was determined using real-time PCR. The *iNOS* level was significantly increased after LDH was established; however, the *iNOS* level was significantly lower in the ILS and OS groups than that in the control group (Figure 3A). In addition, the expression levels of inflammation-related genes, such as *IL-1*β and *TNF-*α, were significantly elevated in the control group compared to the normal group. In contrast, these genes were significantly and dose-dependently decreased in the ILS and OS groups; however, they were not different in the different administration routes of Shinbaro 2 in LDH rats (Figures 3B, C). Furthermore, the western blot analysis was performed to determine the protein expression levels of iNOS, IL-1β, and TNF-α, which were strongly upregulated in the control group compared to those of the normal group, whereas these genes were dose-dependently suppressed in the OS groups, and the iNOS and IL-1β levels were dramatically suppressed in all doses in the ILS groups. However, the TNF-α level was only decreased in the 20 mg/kg ILS group (Figure 3D).

### 3.4. Effect of Shinbaro 2 on the expression of MMP-9, MMP-13, ADAMTS-5 mRNA, and protein in LDH rats

**Figure 4:**
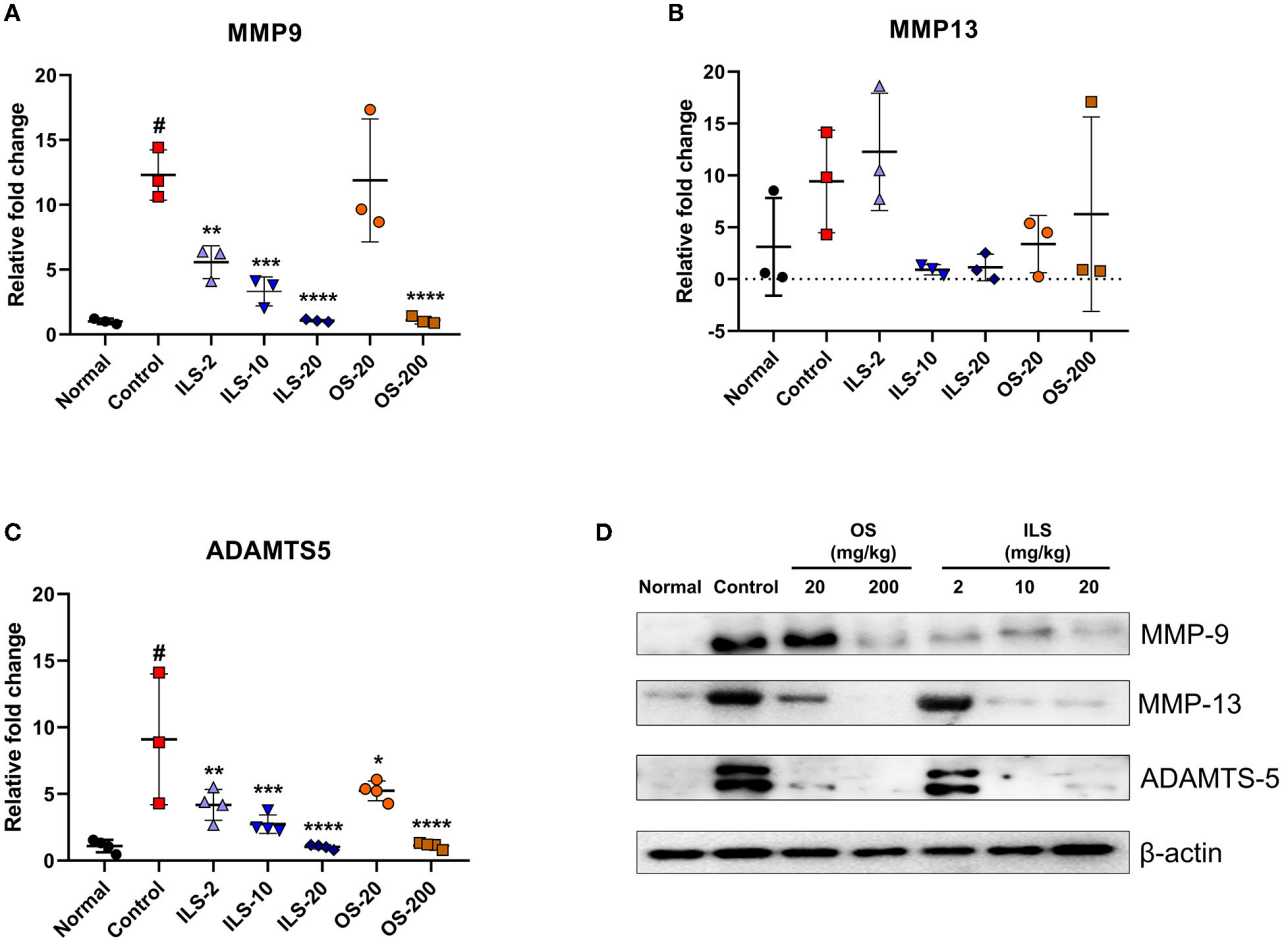
Effect of Shinbaro 2 on the expression of MMP-9, MMP-13, ADAMTS-5 mRNA, and protein in LDH rats. **(A–C)** Gene expressions analyzed with tissue samples after Shinbaro 2 administration with two routes in LDH rats for 14 days: **(A)** *MMP-9*, **(B)** *MMP-13*, **(C)** *ADAMTS-5*, and **(D)** western blot analysis of MMP-9, MMP-13, and ADAMTS-5 protein expression at 14 days. The β-actin image is re-used for an illustrative purpose. Data are expressed as mean ± SEM. Significant differences indicated as ^#^a *p* < 0.001 compared vs. the blank group, *a *p* < 0.05, **a *p* < 0.01, ***a *p* < 0.001, and ****a *p* < 0.0001 vs. the control group were analyzed *via* one-way ANOVA with Tukey's *post-hoc* test.

We investigated disk degeneration-related genes such as *MMP-9, MMP-13*, and *ADAMTS-5*. The expression level of *MMP-9* was significantly higher in the control group than that in the normal group. By contrast, ILS groups significantly decreased the *MMP-9* level in a dose-dependent manner compared to that in the control group, whereas this gene was only significantly decreased in the 200 mg/kg OS group (Figure 4A). However, *MMP-13* was not different between the groups (Figure 4B). ADAMTS-5 plays a critical role in disk degeneration as an extracellular matrix-degrading enzyme. We further examined the *ADAMTS-5* expression level using real-time PCR. The *ADAMTS-5* level was significantly higher in the control group than in the normal group, whereas the ILS and OS groups inhibited this increase in a dose-dependent manner (Figure 4C). Furthermore, we confirmed the change of these factors in the protein level using the western blot analysis. The results revealed a trend similar to that observed in the mRNA level. The protein expression of MMP-9 was higher in the control group than that in the normal group, whereas MMP-9 expression was dramatically reduced overall in all doses after the intramuscular injection of Shinbaro 2 in LDH rats. Furthermore, MMP-13 and ADAMTS-5 expression does not change in the 2 mg/kg ILS group but is dramatically abolished in the 10 and 20 mg/kg ILS groups. In the OS groups, the MMP-9 level was only decreased in the 200 mg/kg OS group, and the MMP-13 and ADAMTS-5 level was dose-dependently decreased in all OS groups (Figure 4D).

## 4. Discussion

Shinbaro 2 has been previously reported to possess anti-inflammatory effects in lumbar spinal stenosis rats (12). The formulation has also shown protective effects against nerve injury and osteoarthritis in preclinical studies (29, 30). Clinical trials found that Shinbaro 2 was efficacious against spine disease, including disk herniation and spinal stenosis, with a generally safe profile under long-term use (27, 31). However, previous studies have not examined the effects of Shinbaro 2 on various molecular markers of LDH *in vivo*. In the present study, the effects of Shinbaro 2 on LDH-induced inflammatory cytokines and disk degeneration were investigated using an *in vivo* LDH rat model. Although many efforts using *in vitro* techniques have been made to understand LDH better, the underlying systemic factors, such as biomechanics, aging, and metabolism, require examination in *in vivo* models, making these studies more translatable for understanding disk degeneration. Disk degeneration in experimental animal models is typically induced by chemical and mechanical stimuli or genetic modifications (32). Human and animal species differ in disk and spine anatomy, tissue composition, physiology, and biomechanics; therefore, results from *in vivo* studies should be interpreted with caution (33). Our study showed that rat activity was impaired after the surgical induction of LDH, whereas Shinbaro 2 administration reversed such impairment. In particular, Shinbaro 2 is applied in two routes to evaluate adverse reactions and effectiveness according to the route of administration. Since drug absorption varies depending on the route of administration, different concentration ranges were set. According to previous studies, oral administration is known to show relatively incomplete absorption compared to intramuscular administration (34). In our study, when administered intramuscularly at a concentration of 20 mg/kg, the pharmacological action was similar to that of the oral administration group at 200 mg/kg. In addition, the pharmacological effect of the intramuscular administration method was immediately and easily measured from a low concentration than the oral administration method. Shinbaro 2 protected against LDH-associated decreases in function without any significant toxicity, evidenced in body weight changes when two routes of administration were applied. Moreover, Shinbaro 2 conserved the structure of the spinal canal, which deteriorated following an LDH induction in the control group. Therefore, the oral administration of Shinbaro 2 preserved the spinal structure and protected against a decline in function without significant toxicity. Currently, chemical irritation resulting in an autoimmune response accounts for many of the radiculopathy symptoms associated with LDH. Herniated disks expose nucleus pulposus antigens, resulting in the recruitment and differentiation of various immune cells. The release of cytokines, such as IL-1β and TNF-α, induces disk degeneration and sciatic pain (35). IL-1β and TNF-α expressions are positively correlated with the perceived pain severity in patients with LDH. The present study showed that IL-1β and TNF-α were significantly elevated after the surgical induction of LDH due to the systemic inflammatory response following surgical events. Although there are individual differences, protein and mRNA expression studies confirmed that Shinbaro 2 administration suppressed IL-1β and TNF-α levels in a dose-dependent manner. Similarly, surgically induced LDH was associated with an increased expression of the pro-inflammatory enzyme iNOS, and the administration of Shinbaro 2 reversed such increases in a dose-dependent manner. MMPs are endopeptidases that can degrade extracellular matrix proteins (36). The exact role of MMPs in disk herniation remains elusive; however, two distinct hypotheses have been proposed. First, increased MMPs compromise the integrity of the surrounding soft tissue, thus contributing to the pathogenesis of disk herniation. Second, herniated disks lead to local inflammation, which promotes the chemotaxis of immune cells. During the process, the secretion of MMPs is elevated to support the infiltration of immune cells (37). The analysis of the *ex vivo* human disk tissue showed that these events are likely to occur in a sequence. In the present study, Shinbaro 2 suppressed the inflammatory responses from surgical auto-transplantation of the coccygeal disk. Consistent with previous studies, MMP secretion was downregulated at the mRNA and protein levels after Shinbaro 2 administration. In addition, ADAMTS is an MMP-related enzyme that can induce disk degeneration by specifically degrading the extracellular matrix. Previous studies have shown that degenerated or herniated IVDs increase IL-1β and upregulate the expression of ADAMTS, which is responsible for disk degeneration by increasing IL-1β expression. Based on our finding that the ADAMTS-5 activity in response to Shinbaro 2 administration was downregulated with decreased IL-1β expression, we found that Shinbaro 2 may be involved in the process of disk degeneration, which is closely related to ADAMTS-5. However, to further validate its effectiveness, additional clinical and preclinical studies are warranted in future. Furthermore, although a chemical composition analysis was performed through the high-performance liquid chromatography analysis of each component for four herbal medicines additionally included in Shinbaro 2 and six indicator biological components of GSCB-5 such as cimifugin for *Ledebouriellae Radix*, 20-hydroxyecdysone (0.311–0.312 mg/g) for *Achyranthis Radix*, acanthoside D (0.577–0.578 mg/g) for *Acanthopanacis Cortex*, onitin-4-O-β-D-glucopyranoside for *Cibotii Rhizoma*, genistin (0.0426-0.0427 mg/g) for *Glycine Semen*, and geniposide (0.431–0.432 mg/g) for *Eucommiae Cortex* (29), and aesculin, caffeic acid, cimifugin, uracil, and adenosine for *Ostericum koreanum* (38), ferulic acid, Z-ligustilide, butylidenephthalide and various polysaccharides for *Angelica pubescens* (21), paeoniflorin that is one of the main bioactive components of *Paeonia albiflora* (39), and 12 compounds for *Scolopendra subspinipes* (40), the active ingredients of Shinbaro 2 have not been directly confirmed. Another limitation of our study was that it was difficult to identify the main component controlling LDH-induced low back pain and radiating pain of the Shinbaro 2, which is a new prescription medicine based on GCSB-5. Therefore, further studies are needed on the detailed molecular mechanism of Shinbaro 2 and the anti-inflammatory properties of its active ingredients.

## 5. Conclusion

The present findings demonstrated that Shinbaro 2 downregulates the LDH-induced expression of inflammatory mediators, pro-inflammatory cytokines, and disk degeneration-related factors. Therefore, Shinbaro 2 administration restores a normal level of behavioral activity, originally disabled by the surgical induction of LDH. Overall, Shinbaro 2 suppresses the inflammation response at LDH sites, exerting a protective effect in LDH rats, thus scientifically verifying the therapeutic effect of LDH.

## Data availability statement

The raw data supporting the conclusions of this article will be made available by the authors, without undue reservation.

## Ethics statement

The animal study was reviewed and approved by Seoul National University Institutional Animal Care and Use Committee (IACUC; permission number: SNU201610-2).

## Author contributions

WKK and J-SS: conceptualization. WKK: methodology, software, and investigation. WKK, J-SS, JL, WK, I-HH, and HJP: validation. WKK, HJP, and JYH: formal analysis. S-KL: resources, supervision, project administration, and funding acquisition. WKK and HJP: data curation. WKK and JYH: writing the original draft preparation and visualization. S-KL and JYH: writing, reviewing, and editing. All authors read and approved the final manuscript.
