# Supplementary figures and images for: Effects of the administration of Shinbaro 2 in a rat lumbar disk herniation model
Source: Front Neurol. 2023 Mar 10;14:1044724. doi: 10.3389/fneur.2023.1044724 (PMC10036394; doi:10.3389/fneur.2023.1044724)

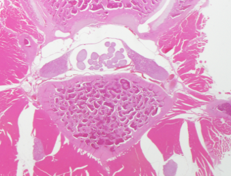

Supplement: Supplementary file 1 [file Data_Sheet_1.ZIP › Raw data/Fig 1/Fig1_Control_H&E.tif]

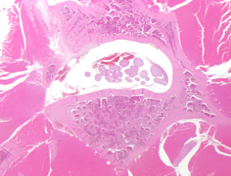

Supplement: Supplementary file 1 [file Data_Sheet_1.ZIP › Raw data/Fig 1/Fig1_Estradiol_H&E.tif]

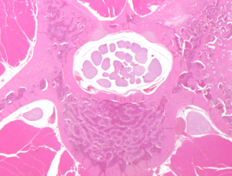

Supplement: Supplementary file 1 [file Data_Sheet_1.ZIP › Raw data/Fig 1/Fig1_ILS-20_H&E.tif]

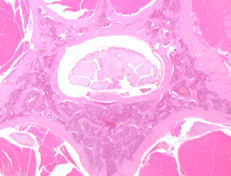

Supplement: Supplementary file 1 [file Data_Sheet_1.ZIP › Raw data/Fig 1/Fig1_Normal_H&E.tif]

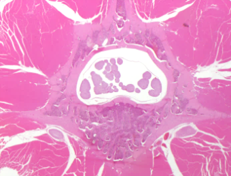

Supplement: Supplementary file 1 [file Data_Sheet_1.ZIP › Raw data/Fig 1/Fig1_OS-20_H&E.tif]

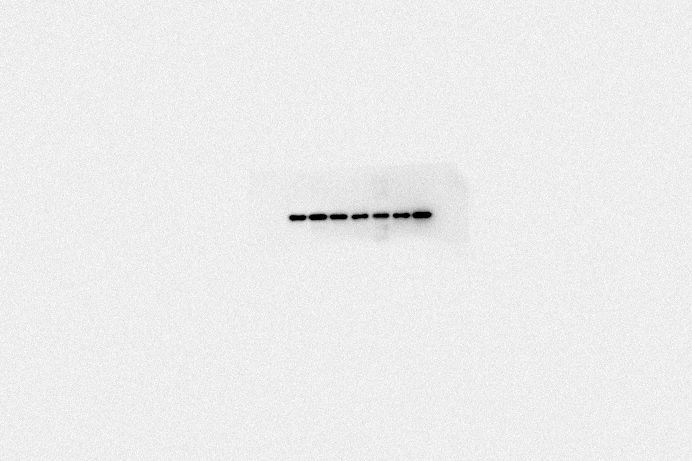

Supplement: Supplementary file 1 [file Data_Sheet_1.ZIP › Raw data/Fig 3/Fig3_actin.tif]
